# The influence of solar-modulated regional circulations and galactic cosmic rays on global cloud distribution

**DOI:** 10.1038/s41598-023-30447-9

**Published:** 2023-03-06

**Authors:** Vinay Kumar, Surendra K. Dhaka, Matthew H. Hitchman, Shigeo Yoden

**Affiliations:** 1 Radio and Atmospheric Physics Lab, Rajdhani College, University of Delhi, New Delhi, India; 2 Department of Atmospheric and Oceanic Sciences, University of Wisconsin–Madison, Madison, Wisconsin USA; 3 Institute for Liberal Arts and Sciences, Kyoto University, Kyoto, Japan

**Keywords:** Climate sciences, Atmospheric science, Atmospheric dynamics

## Abstract

The influence of solar forcing and Galactic Cosmic Rays (GCR) ionization on the global distribution of clouds is investigated using 42 years ERA-5 data (1979–2020). In the mid-latitudes over Eurasia, GCR and cloudiness are negatively correlated, which argues against the ionization theory of enhanced cloud droplet nucleation due to increased GCR during minima in the solar cycle. In the tropics, the solar cycle and cloudiness are positively correlated in regional Walker circulations below 2 km altitude. The phase relationship between amplification of regional tropical circulations and the solar cycle is consistent with total solar forcing, rather than modulation of GCR. However, in the intertropical convergence zone, changes in the cloud distribution are consistent with a positive coupling with GCR in the free atmosphere (2–6 km). This study opens some future challenges and research directions, and clarifies how atmospheric circulation at the regional scale can help in understanding solar-induced climate variability.

## Introduction

Coupling between space and the earth’s atmosphere is a mysterious topic in the scientific community. Our climate system receives energy from the Sun, which can be modulated by variation in the activity of the Sun over long time scales. Solar output exhibits clear variability in the 8–12 year range, with an average 11 year solar cycle. The strength of the solar cycle is observed to vary at decadal and 100-year time scales^1,2^, and evidence for variability at 1000-year time scales is seen in the geological record^3^. The flux of galactic cosmic rays (GCR) reaching the earth from outside of the solar system is observed to vary by about 15%^1^ across the solar cycle, with increased GCR during times of weak solar activity. It has been hypothesized that increased GCR can lead to enhanced cloud condensation nuclei (CCN) and aerosol and thereby possibly increase cloud cover^4–6^. The existence of any viable relationship between GCR and clouds would be an important factor in our weather and climate system, since clouds modulate the Earth’s radiative budget. Indeed, some investigations have shown a significant relationship between solar-modulated GCR flux and cloud cover^7–9^. Nearly 2–3% changes were observed in the global averaged monthly structure of low cloud cover (LCC) in International Satellite Cloud Climatology Project (ISCCP) observational data for the period 1980–1995^7^ and 1983–2005^10^. However, there are some serious scientific questions regarding the calibration techniques of ISCCP data for long terms studies^11–13^, which would undermine the LCC-GCR linkage hypothesis^14^. Some studies have suggested that there is a solid correlation between GCR and LCC^15,16^, while others do not support this linkage^17–19^. Some other studies have found that variations in medium cloud cover (MCC) and high cloud cover (HCC) are also correlated with changes in solar radiation^20,21^.

The Intergovernmental Panel on Climate Change (IPCC) Fifth Assessment Report (AR5)^22^ of Working Group 1 (WG1) concluded that there is no association between GCR and cloudiness. Since the AR5, the linkage between GCR and new particle formation also has been tested in the CERN CLOUD (Cosmics Leaving Outdoor Droplets) chamber experiment^22–25^^.^ On the basis of the CLOUD experiment, Gordon et al.^25^ found that GCR-induced new CCN concentration for low-clouds differed by 0.2–0.3% between solar maximum and solar minimum, which is relatively a very small variation in the atmospheric ion concentration over the centennial time scales^26^. It is therefore unlikely that cosmic ray intensity can affect present-day climate via nucleation theory^22,24,27,28^. Hence, on the basis of the key laboratory, theoretical and observational evidence, the recent IPCC Sixth Assessment Report AR6^29^ of WG1 further reinforced that there is no robust association between GCR and cloudiness.

However, cloud cover varies strongly in latitude, with a large seasonal cycle within 40°S to 40°N, which is driven by a continental-scale contrast between land and oceans and the Earth’s revolution around the Sun^30,31^. Furthermore, the geographical distribution and intensity of clouds are modulated by tropospheric circulations on interannual to intraseasonal scales, including the El Niño Southern Oscillation (ENSO)^32,33^, Walker Circulations (WC)^34,35^, and the Madden–Julian Oscillation (MJO)^36^. At middle and high latitudes, clouds are influenced by synoptic-scale weather systems (cyclones and troughs) and the North Atlantic oscillation (NAO)^37^. Some investigators have suggested that the GCR-cloud linkage may exist for a particular solar cycle due to dynamical phenomena such as ENSO^4,38,39^, or by a response to volcanic activity^4^. The ENSO effect on clouds can dominate over GCR effects in some regions, such as in the Eastern Pacific sector^40^. Convection may be important in amplifying the solar signal in the HCC in the Pacific basin via ocean–atmosphere positive feedbacks^21^. Other studies have pointed out that in the lower troposphere the solar/GCR signal was stronger in regions where warm clouds formed^41^, and that the season and regional-scale orography can also be a crucial factor for an imprint of the solar cycle in cloud cover^42^. Solar-modulated GCR signals in cloudiness may occur indirectly via influences on the troposphere dynamics of extratropical cyclones and troughs that formed the cloud field^43^.

These studies indicate that the formation and variation of cloud cover is a nonlinear complex system, and the GCR-cloud linkage may not be noticeably straightforward. There is a possibility that over a particular region, atmospheric circulations may act as a positive or negative feedback for GCR-cloud linkage. This study highlights the importance of regional atmospheric circulations for making changes associated with the solar modulated natural phenomena prevalent in the cloud cover of our climate system. Here we seek to identify the linkage of atmospheric circulations in solar-modulated GCR on cloud cover using composite differencing between periods of high and low intensity GCR (hereinafter GCR max and GCR min) in the most recent ERA-5 reanalysis data of satellite era from 1979 to 2020. The GCR record is also valuable as a proxy for variability in solar emission, which can have a direct effect on atmospheric heating, circulation, and cloud formation, where a GCR minimum corresponds to a more active sun.

## Results

### Global mean results

**Figure 1:**
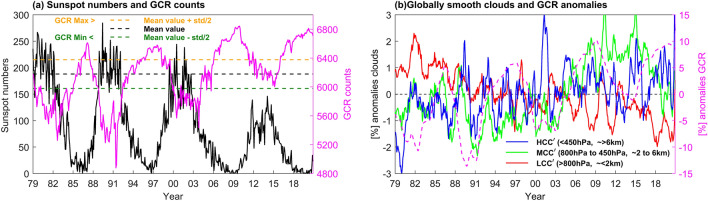
(**a**) Monthly time variation of sunspot number (black curve) and GCR count (pink curve) for the period 1979–2020. The black dashed line is the time mean value of GCR counts, while the gold and green dashed lines indicate one-half standard deviation above and below the mean, defining groups of maximum and minimum GCR, respectively. (**b**) The red, green, and blue lines represent the global mean, twelve month smoothed cloud anomalies (in %) of the LCC$\boldsymbol{^{\prime}}$, MCC$\boldsymbol{^{\prime}}$ and HCC$\boldsymbol{^{\prime}}$, respectively, while the pink dashed line with right side y-axis represents the GCR$\boldsymbol{^{\prime}}$ anomalies (in %).

Time series of solar sunspot number and GCR are shown for the period 1979–2020 in Fig. 1a. A correlation coefficient of − 0.82 is indicated for these two time series. Due to continuous weakening of the solar cycle during this period (Fig. 1a), an increasing trend on the order of 15% is observed in GCR′ anomalies (single prime on superscript indicates de-seasonalized data, see data). This increase is nearly equal to the variation in the GCR intensity within the same solar cycle from maximum to minimum. Time series of global mean LCC$\boldsymbol{^{\prime}}$, MCC$\boldsymbol{^{\prime}}$, HCC$\boldsymbol{^{\prime}}$, and GCR$\boldsymbol{^{\prime}}$ anomalies are shown in Fig. 1b, where a 12-month running mean filter has been applied for the smoothness. The time series of LCC$\boldsymbol{^{\prime}}$ and GCR$\boldsymbol{^{\prime}}$ are anticorrelated, with a correlation coefficient (CC) of − 0.56 ± 0.03, while variations in GCR$\boldsymbol{^{\prime}}$ are positively correlated with MCC$\boldsymbol{^{\prime}}$ and HCC$\boldsymbol{^{\prime}}$, with CCs of + 0.42 ± 0.04, and + 0.26 ± 0.04, respectively (Fig. 1b). The modest negative correlation between GCR′ and LCC$\boldsymbol{^{\prime}}$ is inconsistent with the positive GCR-LCC linkage observed in a previous study^9^. Possible reasons for this difference is that the uppermost pressure level for defining LCC was taken as 680 hPa, which includes part of the free atmosphere above the atmospheric boundary layer (ABL). In this study, pressures corresponding to LCC are below the 800 hPa level, which focuses more cleanly on the ABL. Note that the period of time covered is also longer in the present study.

### Zonal-mean and zonally asymmetric results

**Figure 2:**
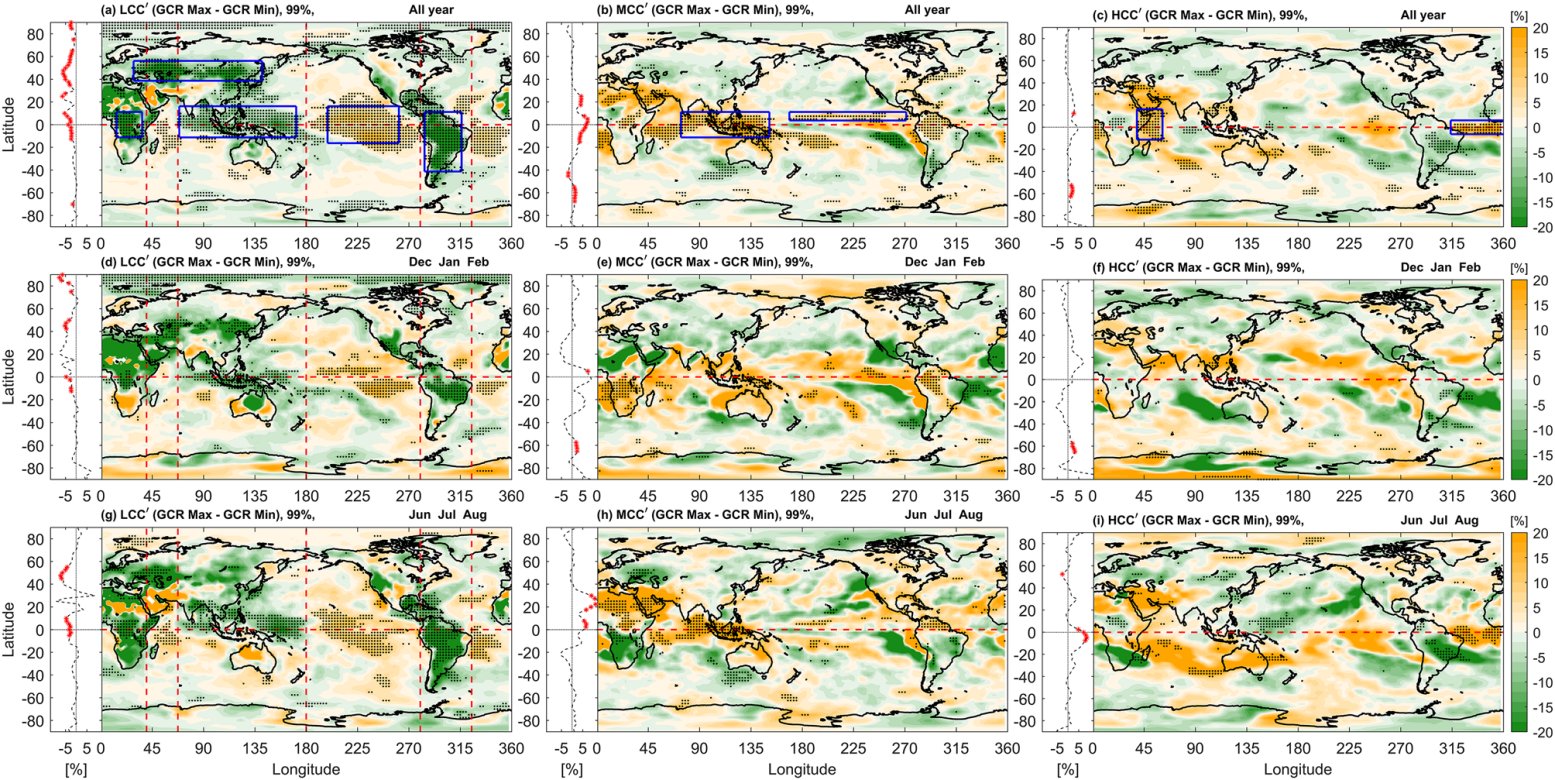
Plan views of composite differences between the GCR max—GCR min groups. From left to right, columns represent the LCC$\boldsymbol{^{\prime}}$, MCC$\boldsymbol{^{\prime}}$, and HCC$\boldsymbol{^{\prime}}$ anomalies respectively. The top row, (**a**–**c**) is for all year data, middle row, (**d**–**f**) for DJF data, and bottom row, (**g**–**i**) for JJA data. Black dots highlight the areas where statistical significance exceeds 99% in the composite difference. The associated plot on the left in dashed lines with each panel presents the zonal mean structure, and the red star markers indicate the latitudinal zone where composite differences exceed 99% statistical significance. The red color dashed lines over the LCC panels (**a**,**d**,**g**) represent the ascending/descending branch of the WC. The blue rectangular boxes are the selected domains for the time series analysis shown in Fig. 3.

The solar cycle influence on cloud cover is investigated in zonal mean and zonally asymmetric variations to look for any regional dependences. Analysis of zonal mean and zonally asymmetric variations with GCR is based on composite difference analysis between the GCR max and GCR min groups (Methods) for all years, analyzed separately for boreal winter (December, January, and February; DJF), and summer (June, July, and August; JJA) seasons (Fig. 2). This allows for investigating the possible influence of regional dynamical motions on the solar cycle-cloud linkage. The significant patterns exist at the continental-scale, related to topography and the distribution of land and sea in the latitude range (20°S to 90°N).

The composite difference for seasonal LCC$\boldsymbol{^{\prime}}$ exhibits significant dipole patterns in the tropical and subtropical regions (Fig. 2a, d and g). A strong relationship can be seen between the longitudinal range of positive/negative value of dipole patterns and the descending/ascending branch of the WC (see Fig. S1 in supportive information (SI)). The broadest significant dipole pattern is associated with the broadest cell of the WC, extending from the Indo-Indonesian region across the Pacific Ocean. Also, seasonal modulations in the significant dipole patterns and the WC exhibit a clear resemblance to each other. These patterns shift northward and westward during boreal summer (compare Fig. 2d and g), along with the seasonal shift in the Maritime Continent, a region of chronic deep convection (Fig. S1d,f). The reduction in the intensification and magnitude of upward motion over Indonesia in the band 5$^\circ$S-5$^\circ$N from DJF to JJA (Fig. S1c,e), is due to the northwestward shift of convection toward Southeast Asia. A similar relationship between the significant dipole patterns and the WC can be seen during the equinoctial seasons March, April, and May (MAM) and September, October, and November (SON) (Figs. S2 and S3). It is noteworthy that significant patterns over Indonesia in the band 5$^\circ$S-5$^\circ$N are sparse in all years and are absent in JJA, and SON.

In midlatitude regions, a zonally-elongated band of significant negatively correlated relationship between LCC$\boldsymbol{^{\prime}}$ and GCR$\boldsymbol{^{\prime}}$ is found in the composite difference over Eurasia (40°N-55°N, 30°E-140°E), in all year and seasonal data. MCC$\boldsymbol{^{\prime}}$ and HCC$\boldsymbol{^{\prime}}$ composite difference patterns over this region show a similarly-shaped negative region. However, values are not very statistically significant and amplitudes are weak. A negative region in LCC$\boldsymbol{^{\prime}}$ can also be observed over the southwestern United States. Although not statistically significant, this supports the previous study^44^ that cloud variations over the U. S. are anti-correlated with GCR and in phase with the solar cycle. In the polar region (> 75°N), a significant negative region of LCC$\boldsymbol{^{\prime}}$ is found, but only during boreal winter (Fig. 2d).

Composite difference of MCC$\boldsymbol{^{\prime}}$ demonstrate robust significant positive patterns over the eastern Pacific region in all years data (Fig. 2b). The climatological annual cycle of precipitation (1979–2020), which is used as a proxy of the Inter-Tropical Convergence Zone (ITCZ) is shown in SI (Fig. S1). On the seasonal scale (DJF to JJA), the ITCZ shifts from the Southern to Northern Hemisphere (Fig. S1d,f). The MCC$\boldsymbol{^{\prime}}$ difference pattern exhibits a statistically significant positive region near the ITCZ, flanked by an eastward-opening chevron-shaped negative region extending over the subtropical eastern Pacific. Significant positive regions for MCC$\boldsymbol{^{\prime}}$ are also seen over Africa and Indonesia (Fig. 2b,e,h), again migrating with the seasonal shift in location of chronic deep convection. The two equinox seasons (MAM, and SON) also show significant patterns for MCC$\boldsymbol{^{\prime}}$ near the ITCZ (Figs. S2 and S3). Zonal mean structures also show significant patterns in the latitude range of the ITCZ (20°S to 20S°N), with a peak that lies at 7.5°N in all years, the center for ITCZ in zonal mean structure. For HCC$\boldsymbol{^{\prime}}$, patterns are somewhat similar to MCC$\boldsymbol{^{\prime}}$, but with reduced significance (Fig. 2c,f,h). We also performed statistical analysis after removing months with significant ENSO amplitude and obtained similar patterns (Fig. S4).

### Regional time series

**Figure 3:**
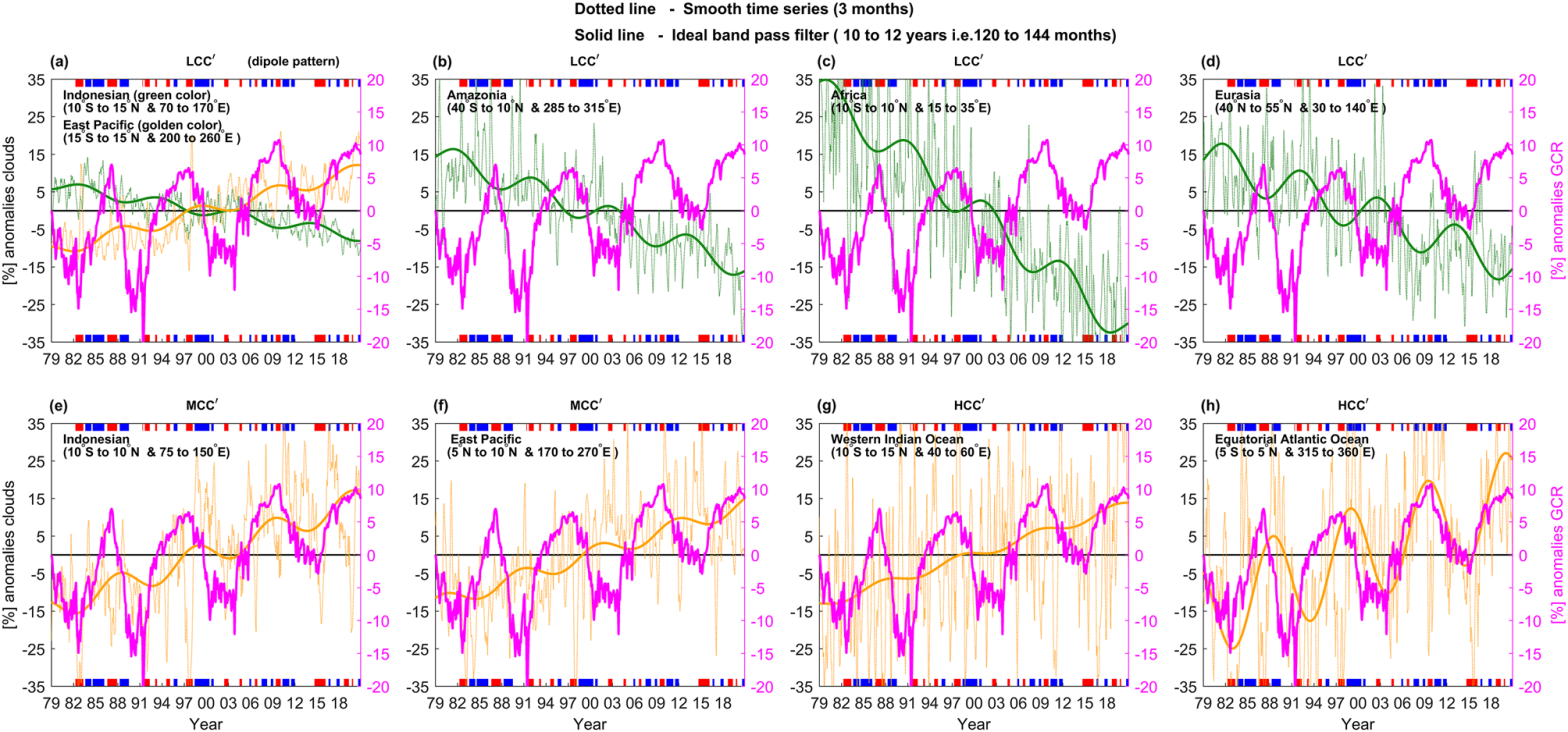
Time variation of cloud cover anomalies for selected regions shown in Fig. 2. In all figures the dotted line represents three month-averaged cloud cover anomalies, while the solid line represents ideal band-pass filtered data, allowing the signal between 10 and 12 years (120–140 months) in the monthly mean data. The golden (green) of dotted lines represent areas which show significant positive (negative) anomalies in the composite difference analysis. (**a**) LCC$\boldsymbol{^{\prime}}$ anomalies for the Indonesian and East Pacific sector dipole pattern (Fig. 2a). (**b**–**d**) LCC$\boldsymbol{^{\prime}}$ anomalies for the Amazonia, African, Eurasia regions (Fig. 2a). (**e**) and (**f**) MCC$\boldsymbol{^{\prime}}$ anomalies for the Indonesian and East Pacific sectors. (**g**) and (**h**) HCC$\boldsymbol{^{\prime}}$ anomalies for the Western equatorial Indian Ocean and equatorial Atlantic Ocean. The strips at the base and top with red and blue colors indicate El Niño and La Niña periods, respectively.

In order to further investigate particular regions of interest, we analyzed time series over selected domains which exhibit significant patterns in the composite difference (highlighted with blue rectangles in Fig. 2a, b and c). A Fast Fourier Transform (FFT) was applied to the monthly mean data for each rectangle to detect the periodicity. A time period of 10.5 years (126 months) with 99% significant level is found in time series of all rectangles. Hence the monthly data of each rectangle is passed through an ideal band-pass filter which retains only periodicities of 10–12 years, corresponding to 120 to 144 months. Further, a forward three-month running mean is applied to each time series in each rectangle to smooth monthly fluctuations, and these are compared with time series resulting from an ideal band-pass filtered data, as shown in Fig. 3. The resulting GCR$\boldsymbol{^{\prime}}$ anomalies are also shown in Fig. 3, along with the base and top strips which reflect El Niño (red) and La Niña (blue) periods, defined by when the three month forward-running average ENSO index exceeds |± 0.5 K|. Table 1 summarizes the CCs between ideal band-pass filtered and GCR$\boldsymbol{^{\prime}}$ anomaly time series for these selected zones.

**Table 1:** The correlation coefficient between ideal band-pass filtered and the GCR$\boldsymbol{^{\prime}}$ anomaly series for selected domains which exhibited significant patterns in the composite difference.

Cloud Level	Region	Correlation coefficient ± Standard error
LCC	Indonesian	– 0.67 ± 02
	East Pacific	0.67 ± 02
	Amazonia	– 0.72 ± 02
	Africa	– 0.69 ± 02
	Eurasia	– 0.78 ± 02
MCC	Indonesian	0.70 ± 02
	East Pacific	0.52 ± 03
HCC	Western Indian Ocean	0.66 ± 02
	Equatorial Atlantic Ocean	0.68 ± 02

Time series, trends, and periodical signal in the filtered data for the dipole pattern over the Indonesian and East Pacific sectors exhibit anti-correlation in the LCC$\boldsymbol{^{\prime}}$ anomalies (Fig. 3a). Signals with a period of 10 to 12 years can be seen in the filtered data of both regions. The East Pacific sector shows an in-phase relationship between cloudiness and GCR$\boldsymbol{^{\prime}}$ (+ 0.67 CC), while the Indonesian sector is anti-phased (− 0.67 CC), although both regions tend to lag maxima in GCR$\boldsymbol{^{\prime}}$. It is apparent from the analysis that both the linear trends and the periodical signal may be induced by solar forcing. Greenhouse effects cannot be held accountable for the periodical signal, as it is continuously increasing. Clear positive spikes can be seen in the Pacific time series during extreme El Niño events (~ 1983, 1987, 1998, 2009 and 2015), whereas these became negative in the Indonesian sector. On the other hand, there are no such sharp spikes associated with the La Niña events.

Other regions of significant negative patterns for LCC$\boldsymbol{^{\prime}}$ in the composite difference can be seen over Amazonia, Africa, and Eurasia. All these regions show clear trends and a periodical signal opposite in phase to GCR$\boldsymbol{^{\prime}}$ (Fig. 3b, c and d). ENSO modulations are absent in the time series of these regions. The amplitude of the periodical signal is stronger over the Eurasian sector, in comparison to other regions. However, during this 42-year time span, the LCC in the African region shows the steepest decrement (~ 35%).

Time series, trends, and a signal of ~ 10 to 12 years in the filtered data for MCC$\boldsymbol{^{\prime}}$ anomalies in the Indonesian and East Pacific ITCZ region (defined in Fig. 2b), are shown in Fig. 3e,f). The relationship between MCC$\boldsymbol{^{\prime}}$ and GCR$\boldsymbol{^{\prime}}$ in the Indonesian sector is similar to LCC$\boldsymbol{^{\prime}}$, but is opposite in sign (compare green curve in Fig. 3a and orange curve in 3e), consistent with results from Fig. 2. MCC$\boldsymbol{^{\prime}}$ in the eastern Pacific ITCZ region is antiphased with GCR$\boldsymbol{^{\prime}}$ (Fig. 3f). Strong short-time scale fluctuations in the time series of MCC$\boldsymbol{^{\prime}}$ over the Indonesian region are probably related to ENSO events, where negative spikes occur during El Niño and positive during La Niña events. Spikes in time series of the Pacific sector are rather variable, and do not show any systematic variation with ENSO. Regions of deep tropical convection are influenced by other phenomena such as the Pacific decadal oscillation (PDO) and the MJO. Despite this, a periodical signal still exists in the filtered data, with a time lag in GCR anomalies during each solar modulated GCR cycle.

The relationship between GCR$\boldsymbol{^{\prime}}$ and HCC$\boldsymbol{^{\prime}}$ in the equatorial Western Indian Ocean and equatorial Atlantic Ocean is shown in Fig. 3g,h. The signal in HCC$\boldsymbol{^{\prime}}$ in the Western Indian Ocean is similar to that for MCC$\boldsymbol{^{\prime}}$ but is smaller. A more robust signal can be seen in the equatorial Atlantic Ocean region, with a same-phased relationship between GCR$\boldsymbol{^{\prime}}$ and HCC$\boldsymbol{^{\prime}}$ in both the trend (i.e., anti-phased with solar-cycle periodicity) (Fig. 3h). Clear positive (negative) spikes can also be observed in the Atlantic Ocean time series during extreme El Niño (La Niña) events, which is consistent with previous findings^45^.

## Discussion

The present study investigates possible relationships between the distribution of cloudiness, the solar cycle, and its trend during 1979–2020, using GCR$\boldsymbol{^{\prime}}$ as a proxy which is strongly anti-correlated with total solar activity. Recent global warming trends cannot account for the significant increasing trends (~ 15%) observed in the GCR counts, as they originate from outer space, and it is well-understood that weakening of the solar activity is directly related to intensification in the GCR counts. In fact, GCR modulation is related to solar activity, with relevant factors including the sunspot number, solar polar field strength, heliospheric current sheet, tilt angle, solar wind speed, solar wind dynamic pressure, and the interplanetary magnetic field magnitude and its fluctuation^46,47^. Analysis suggests that both the trends and periodicity observed in cloud cover may be linked with solar forcing. Owing to variations in the sign of this relationship across the globe and in zonal mean quantities due to different localized regional atmospheric circulations, this linkage can be more clearly understood at the regional scale.

**Figure 4:**
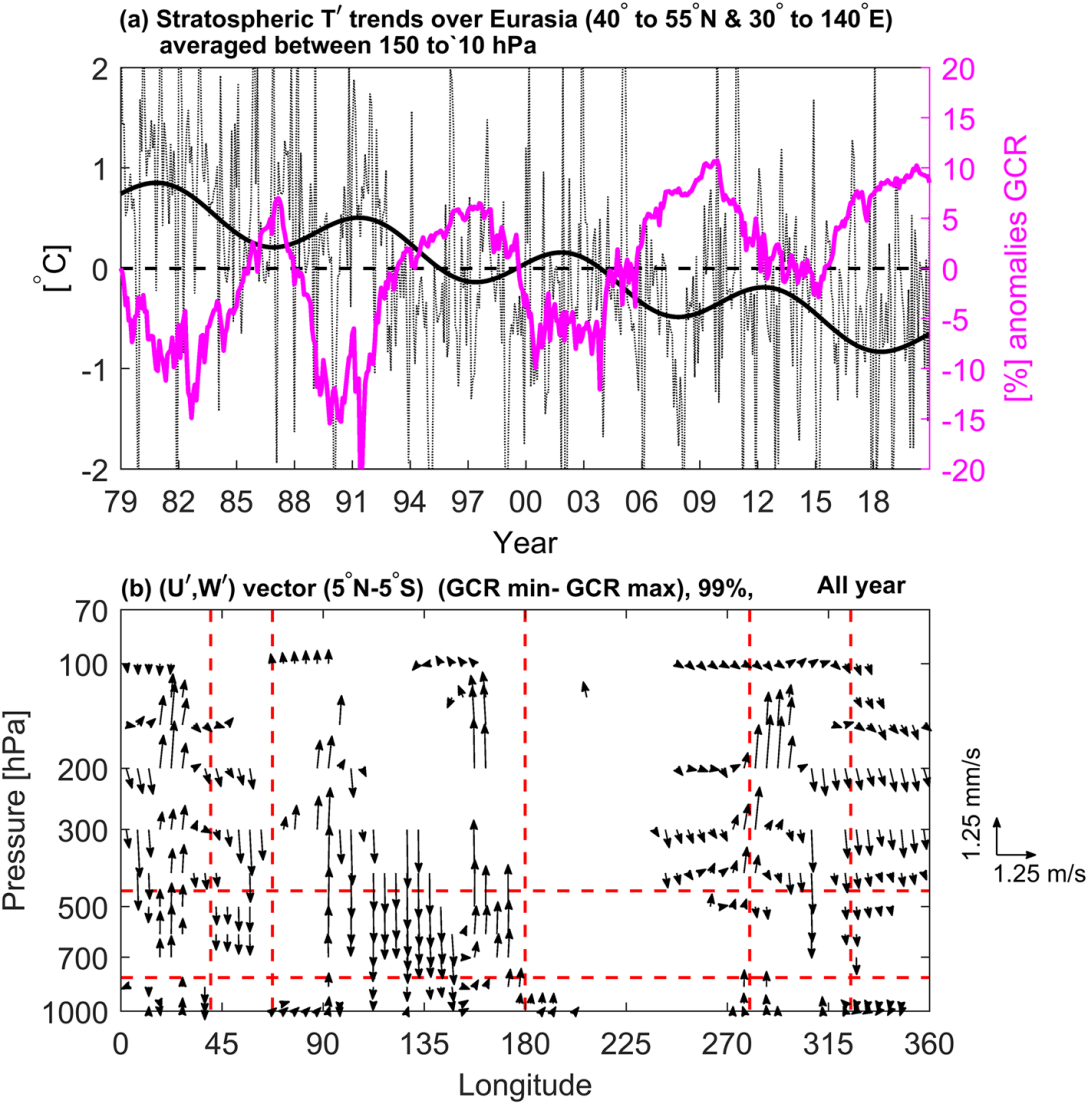
(**a**) Monthly time variation of stratospheric T$\boldsymbol{^{\prime}}$ trends (150–50 hPa, dotted curve) over the Eurasia region, where the sold line represents the ideal band-pass filtered data, which allows the signal between 10–12 years periodicity. GCR anomalies are shown with the purple curve. (**b**) The composite difference (GCR min—GCR max group) of zonal mean (U$\boldsymbol{^{\prime}}$**,** W$\boldsymbol{^{\prime}}$) wind vectors (5°S to 5°N) is plotted, only if either component’s statistical significance exceeds the 99% in the composite difference.

In the midlatitudes over Eurasia, the significant negative GCR-LCC linkage argues against the ionization theory of enhanced cloud droplet nucleation due to increased GCR during minima in the solar cycle. However, much of these results may be consistent with variations in regional circulation strength to total solar forcing. It is possible that small changes in total tropospheric heating can amplify or diminish regional circulations driven by convection^48^. It is also possible that changes in solar heating change the whole general circulation in ways that are not yet well-understood. To explore the solar forcing hypothesis, temperature variations in the extratropical lower stratosphere are compared with GCR as a solar output proxy in Fig. 4a.

Although stratospheric temperatures are subject to dynamical phenomena as well as solar output, especially during winter and spring, Fig. 4a shows that stratospheric temperature variations over the Eurasian sector are correlated with changes in the solar output (anti-phased with GCR). Weakening solar forcing, increasing greenhouse gas loading, and the reduction in ozone over this period of time can each contribute toward the downward trend in stratospheric temperatures. However, a periodical signal seems to be induced by solar forcing, in contrast to greenhouse effects, which should continuously increase without any periodicity. Due to dynamical vertical coupling, solar-induced heating perturbations in the lower stratosphere can have an impact on the tropospheric circulation, including jet streams, storm-tracks, and eddy-induced meridional circulations, even without any direct forcing below the tropopause^49^. Changes in the tropospheric circulation can, in turn, play an important role in creating regional cloud anomalies. Such a modulation from above can be referred to as a “top-down” effect.

In the tropics, a possible mechanism for forcing systematic dipoles patterns in LCC associated with the WC can be understood in terms of solar forcing. The WC should be stronger when the solar cycle is near its maximum, which would be consistent with an enhanced response directly due to solar heating. Such modulation by changes in solar heating directly in the troposphere may be referred to as a “bottom-up” effect. Figure 4b shows that all cells of the WC are strengthened during solar maxima (i.e. weaker during GCR maxima) except for some opposite features over Indonesia (100°E-140°E) which is a highly convective zone whose vertical motions are influenced by other dynamical motions, such as ENSO and the stratospheric QBO^50,51^, and areas of significance are sparse or absent. This supports the idea that in the troposphere there are already enough CCN, so that cloudiness may not be limited by CCN availability. This further suggests that lower level cloudiness is most directly related to the vertical motion field, which is in turn related to the distribution of net heating. This is consistent with the previous results of Wang et al.^52–54^ who showed that SAGE-II detected cloudiness and tropospheric ozone distribution are directly related to the vertical motion field associated with the Hadley cell and Walker circulation (cf. Figures 5 and 6 in Wang et al.^54^). It is interesting that both the solar cycle and its long-term trend seem to be related to changes in tropical cloudiness.

Changes in solar heating and associated changes in regional tropical circulations may also explain the ITCZ/chevron pattern seen in the eastern Pacific for MCC (Fig. 2b,e,h). However, it is possible that increased GCR-induced CCN is a relevant process in the free troposphere (~ 2–6 km) in the eastern Pacific. Note that only the equatorial Atlantic Ocean region (5°N-5°S) demonstrated a significant GCR signal in HCC. However, on the basis of the present analysis, it is difficult to discern possible mechanisms for this GCR signal in HCC. This aspect will be taken up separately. It should also be mentioned that no GCR-cloud correlation is found to be associated with the El Nino Southern Oscillation.

De Mendonca et al.^55^ showed that GCR fluxes are always corrected for pressure and temperature effects, but it is necessary to take the local atmospheric effects into account. They observed an anti-correlation between the temperature variation at the altitude of the maximum charged particle production and temperature at ground level, but this is not valid for the entire globe. It appears clear that conditions on the earth’s surface may be not optimal to understand possible GCR-clouds linkages. This enforces the statistical analysis of the current study that in the lower atmosphere the LCC variations could just be related to effects near the earth surface due to combined effect of solar forcing and localized circulations. The positive statistical correlation of GCR with MCC over the ICTZ and HCC over the Atlantic Ocean may be associated with changes in GCR-induced CCN which increases above the lower troposphere. This suggests that a positive impact of GCR on middle and upper tropospheric cloud cover cannot be ruled out.

These results show that changes in atmospheric circulation must be taken into account in trying to understand climate-solar linkages. The role of solar modulations discussed above should be recognized in relation to their effects on changes in the climate. This study opens up future challenges and research directions regarding solar-induced climate variability, especially the role of regional atmospheric circulations.

## Data and method

### Data

Monthly mean ERA-5 reanalysis data for 42 years over the period 1979–2020 are used for analysis of cloud cover, zonal wind (U), and vertical wind (W). The ERA-5 data set offers several improvements over its predecessor, the ERA-Interim version, as it benefited from a decadal (2006–2016) development in core dynamics, model physics, and data assimilation^56^. ERA-5 outperforms the high resolution regional analysis on a 31-km horizontal resolution which allows for the detailed evolution of weather systems^56^. The ERA-5 data provide an improvement in cloud cover also, and show greater similarity in spatial pattern with satellite observations^57^. Based on pressure levels, ERA-5 cloud data are available in layers as LCC (> 800 hPa ~  < 2 km), MCC (800 hPa–450 hPa ~ 2–6 km), and HCC (< 450 hPa ~  > 6 km). Wind data are available at 37 pressure levels from the surface to 1 hPa. This study explores both zonal mean and zonally asymmetric variations. A single prime superscript for a given variable, X$\boldsymbol{^{\prime}}$, represents its deviation from the mean climatological annual cycle for the 42-year period, i.e. de-seasonalized anomaly. All the results for both the zonal and non-zonal components are presented at 2.5° spatial grid resolution.

The monthly Niño 3.4 index in the region 5°N-5°S, 120°W-170°W is calculated using the Hadley Centre Global Sea Ice and Sea Surface Temperature (HadISST) v1.1 data^58^, which is defined as the de-seasonalized SST anomaly for the 42-year data set. The Niño 3.4 index is used to define periods of El Niño and La Niña whenever it exceeds the threshold values ± 0.5 K (+ El Niño, − La Niña). The data for sunspot number (version 2) are taken from the world data center for sunspot index and long term solar observations (WDC-SILSO), Royal observatory of Belgium, Brussels. GCR data were taken at Oulu (65.05°N, 25.47°E) Finland, operated by the Sodankyla geophysical observatory of the University of Oulu, which is one of the most reliable and stable stations of the world neutron monitoring network^59^, with a vertical geomagnetic cutoff rigidity of ~ 0.8 GeV.

### Methods

The time variation of sunspot numbers (black lines), along with GCR counts (pink line) is shown in Fig. 1a. The intensity of sunspot numbers gradually reduced from the 21st to the 24th solar cycle, with the maximum number of sunspot number in the 24th cycle less than half that of in the 21st cycle (Fig. 1a). This weakening of the solar cycle results in a reduction of the solar activity, hence, a simultaneous increase in GCR counts can be observed. Solar cycle and GCR show a negative correlation coefficient of − 0.82. Due to uneven variation of GCR counts from cycle to cycle, the data set is divided into two groups by introducing the threshold values corresponding to whenever GCR counts are greater (less) than one-half of the standard deviation above (below) the time mean (1979–2020). The thresholds that separate these two groups are shown with golden and green color dashed line (Fig. 1a). Half standard deviation lines added to the mean (data above golden line, and below green line) is useful to show a clear variability in the cloud cover between two extreme values of GCR counts associated with solar cycle. The top group period (above golden line, positive side) is defined as the GCR maximum count group (GCR max), the bottom group period (below green line, negative side) as the GCR minimum count group (GCR min), and rest of the months are referred to as the transition group. The number of months belong to each group are summarized in Table 2, on the annual as well as seasonal basis. A large sample number, with almost equal weighting belong to each group, was obtained for the annual as well as seasonal records. Using a two-sided Student’s t-test, the statistical significance of the composite difference was evaluated in the monthly mean data by assuming two independent samples in each GCR max and GCR min group. Further, to avoid monthly intra-seasonal variability within the cluster of any season, the de-seasonalized anomalies are used for the composite difference analysis (i.e. deviations from the climatological annual cycle). All results for composite difference patterns are discussed at the 99% significant level.

**Table 2:** All year and seasonal sampling in months belongs to each GCR Max and GCR Min groups during All, subdivided into El Niño, La Niña, and Neutral periods for the 42 years 1979–2020.

Data period (1979–2020)	All year	DJF	MAM	JJA	SON
	All El Niño La Niña Neutral	All El Niño La Niña Neutral	All El Niño La Niña Neutral	All El Niño La Niña Neutral	All El Niño La Niña Neutral
GCR Max	183 53 57 73	45 17 20 08	48 12 10 26	46 08 08 30	44 16 19 09
GCR Min	161 35 37 89	43 11 14 18	38 07 09 22	39 09 06 24	41 08 08 25

There may be some decadal bias in the results for composite differences, as the GCR min group does not contain any data from 2006 onward. So, we also performed statistical analysis for the shorter periods 1979–2003 and 1979–1997, when there exits uniformity in data points belonging to different time intervals. Similar significant patterns are observed, which supports the robustness of results from considering the whole data record (1979–2020).

## Supplementary Information

Supplementary Figures.

## Data Availability

All data used in this study are publicly available. The ERA-5 data set is accessible online at https://cds.climate.copernicus.eu/cdsapp#!/home. The HadISST data set is available at https://www.metoffice.gov.uk/hadobs/hadisst/data/download.html. The monthly sunspot numbers and GCR count data are available at https://www.sidc.be/silso/datafiles, and https://cosmicrays.oulu.fi/, respectively.
