# Supplementary material for: The influence of solar-modulated regional circulations and galactic cosmic rays on global cloud distribution
Source: Sci Rep. 2023 Mar 6;13:3707. doi: 10.1038/s41598-023-30447-9 (PMC9988889; doi:10.1038/s41598-023-30447-9)
Supplement: Supplementary file 1 — Supplementary Figures. [file 41598_2023_30447_MOESM1_ESM.pdf]

Supplementary Figures

The influence of solar-modulated regional circulations and galactic cosmic rays on global cloud distribution

Vinay Kumar[1#], S.K. Dhaka[1], Matthew H. Hitchman[2], and Shigeo Yoden[3]

[1] Radio and Atmospheric Physics Lab., Rajdhani College, University of Delhi, India

[2] Department of Atmospheric and Oceanic Sciences, University of Wisconsin–Madison, Wisconsin, USA

[3] Institute for Liberal Arts and Sciences, Kyoto University, Kyoto, Japan

#*Corresponding author*: Vinay Kumar, dabas.vinay@gmail.com

ORCID ID-

Vinay Kumar - https://orcid.org/0000-0003-3582-7625

S. K. Dhaka - https://orcid.org/0000-0002-0812-752X

Matthew H. Hitchman - https://orcid.org/0000-0001-9015-0579

Shigeo Yoden - https://orcid.org/0000-0003-1663-4588

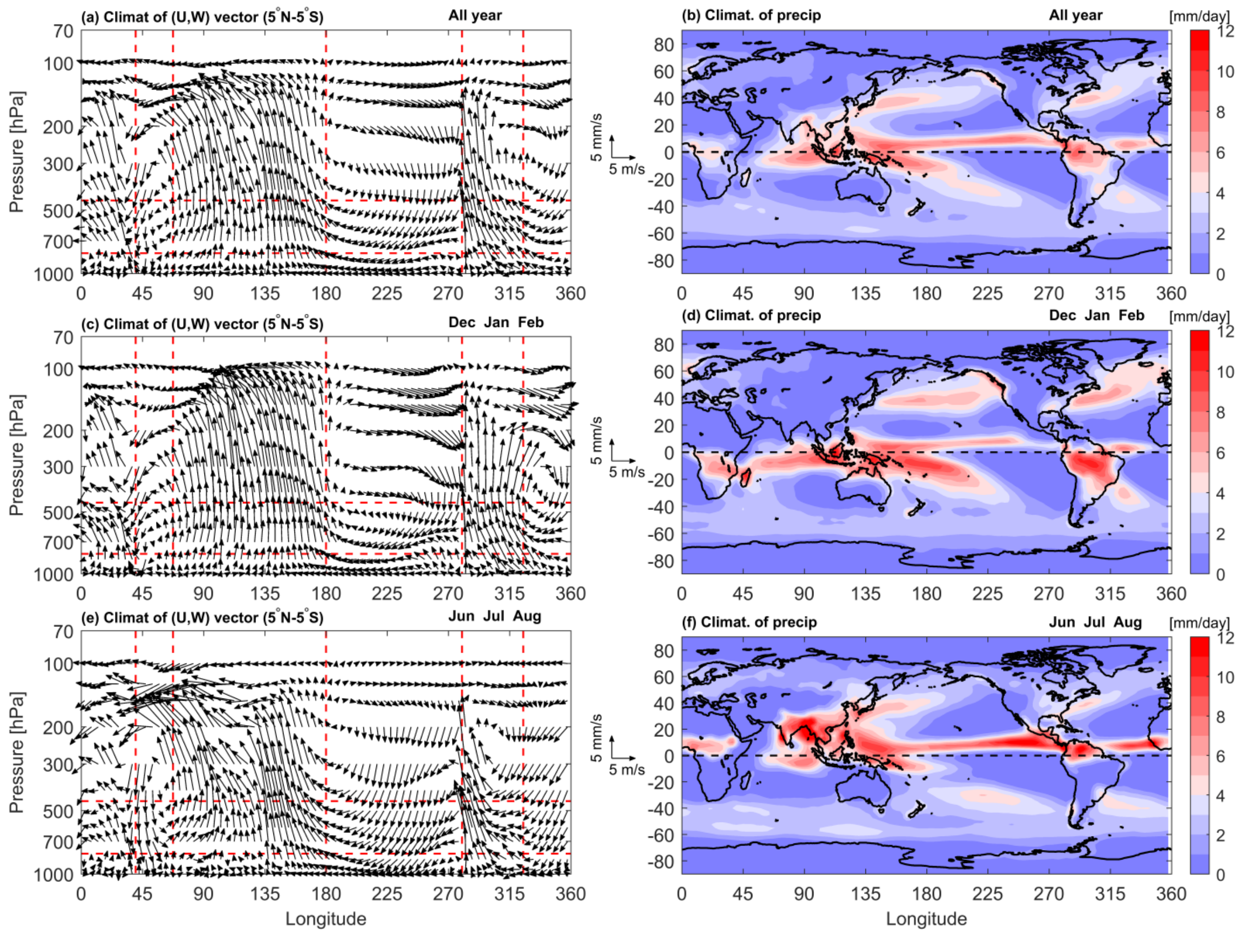

Fig.S1 1st column represents the 42 years (1979-2020) climatology of the (U, W) wind vectors averaged in the latitude domain 5°S to 5°N. 2nd column represents the climatology cycle of precipitation at 2.5° x 2.5° (latitude x longitude) grid map for the same time period. From top to bottom, rows are for all year, DJF, and JJA data, respectively.

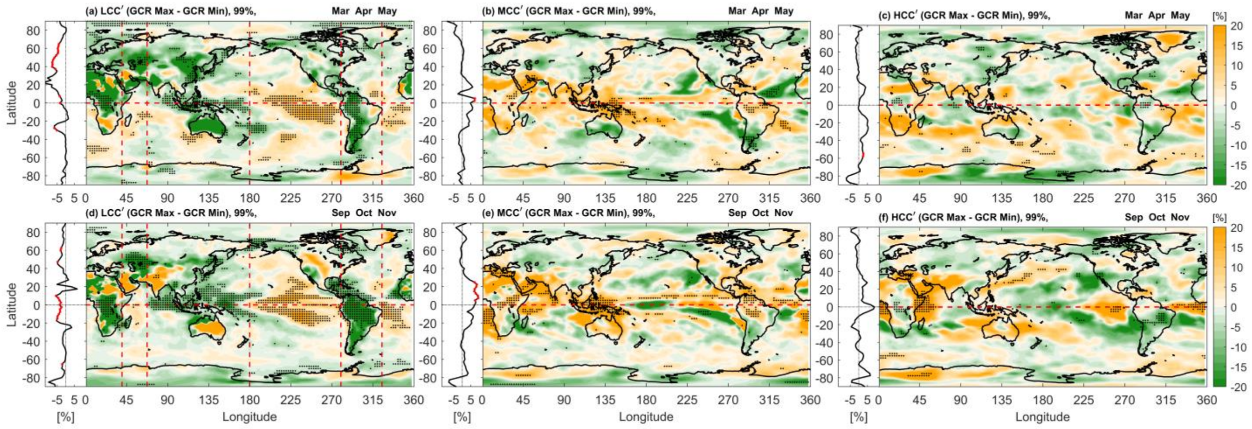

Fig. S2 Same as Fig. 2, but for MAM and SON.

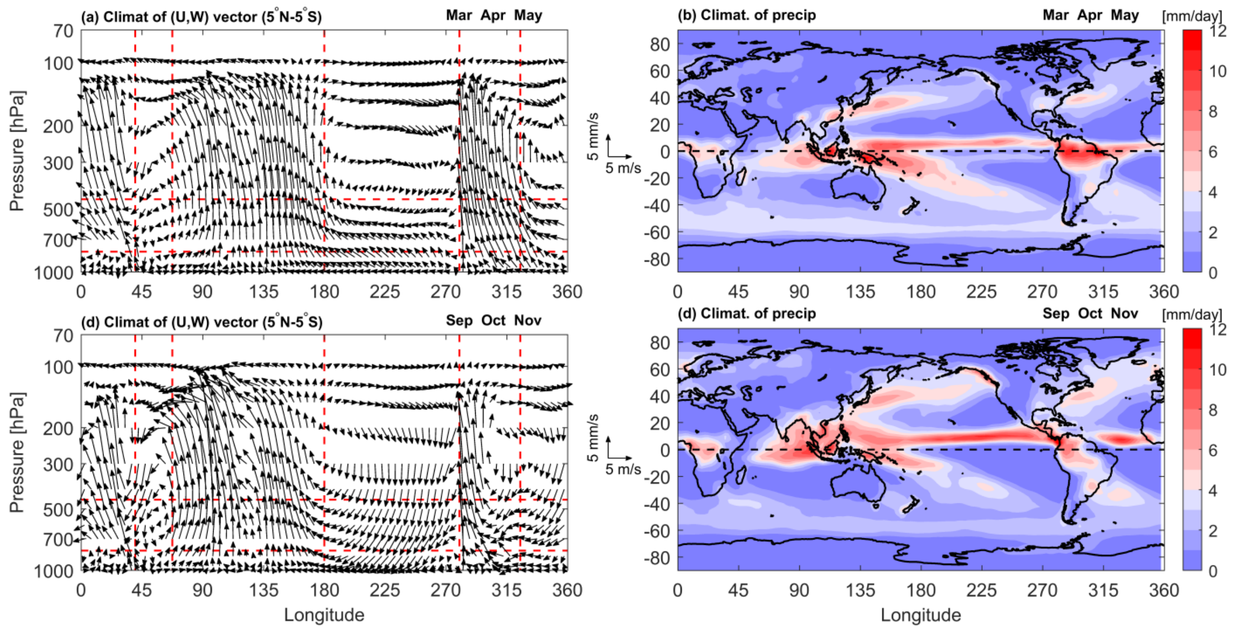

Fig. S3 Same as Fig. S1, but for MAM and SON.

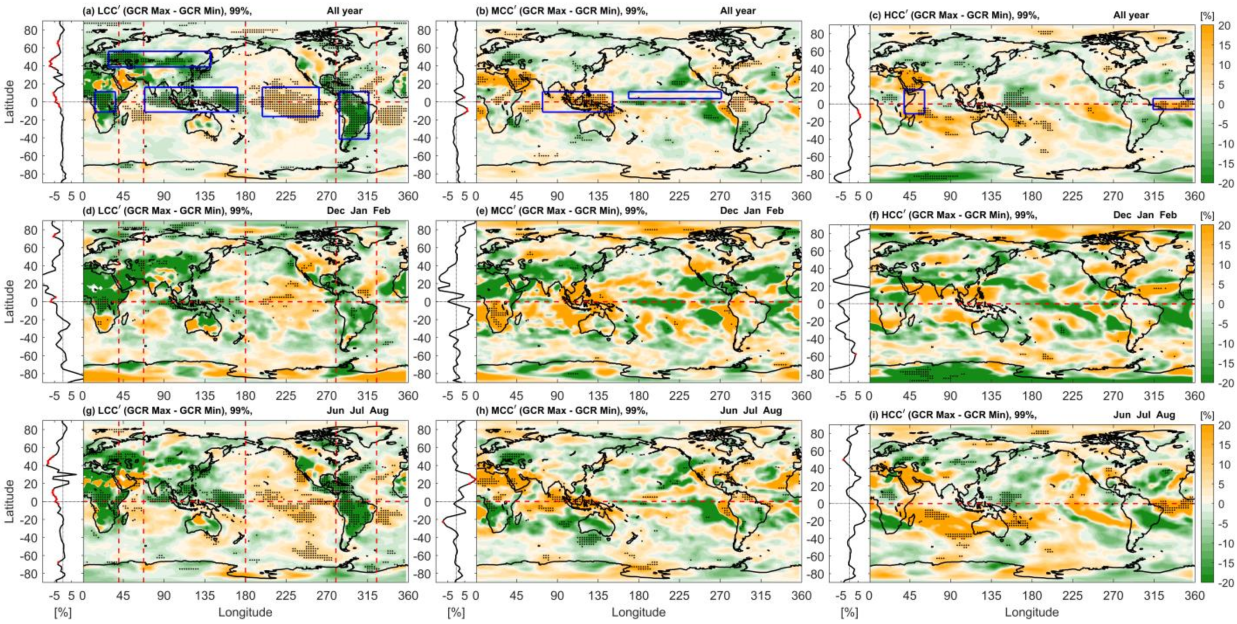

Fig.S4 Same as Fig.2, after excluding the El Niño and La Niña periods.
